# Prevalence of post-vaccine side effects among COVID-19 immunized community of Southern Pakistan

**DOI:** 10.1371/journal.pone.0285736

**Published:** 2023-05-23

**Authors:** Ali Qureshi, Syed Azhar Syed Sulaiman, Wajiha Rehman, Asim Mehmood, Sumaira Idrees, Narendar Kumar

**Affiliations:** 1 Discipline of Clinical Pharmacy, Universiti Sains Malaysia, Pulau Penang, Malaysia; 2 Faculty of Pharmacy, University of Sindh, Jamhoro, Pakistan; 3 Faculty of Public Health and Tropical Medicine, Department of Health Informatics, Jazan University, Jazan, Saudi Arabia; Kwame Nkrumah University of Science and Technology, GHANA

## Abstract

**Background:**

The response to the vaccine may vary among individuals. Hence, it is important to know how often individuals experience side effects after immunization against COVID-19.

**Objective:**

This study aimed to assess the incidence of side effects following COVID-19 vaccination across different vaccine recipients in Southern Pakistan and identify the potential factors associated with these side effects in the population.

**Methods:**

The survey was conducted across Pakistan through Google-forms Links from August to October 2021. The questionnaire included demographic information and COVID-19 vaccine information. Chi-square (*x*^2^) was performed for comparative analysis to check the significance level with *P <0*.*05*. The final analysis included 507 participants who had received COVID-19 vaccines.

**Results:**

Of the total 507 COVID-19 vaccines recipients, 24.9% received CoronaVac, 36.5% received BBIBP-CorV, 14.2% received BNT162b2, 13.8% received AZD1222, and 10.7% received mRNA-1273. The most prominent side effects after the first dose were fever, weakness, lethargy, and pain at the site of injection. Moreover, the most commonly reported side effects after the second dose were pain at the injection site, headache, body ache, lethargy, fever, chills, flu-like symptoms, and diarrhea.

**Conclusion:**

Our results suggested that the side effects due to COVID-19 vaccination can vary between the first and second doses and type of COVID-19 vaccine. Our findings suggest continuing monitoring of vaccine safety and the importance of individualized risk-benefit assessment for COVID-19 immunization.

## Introduction

A vaccine is a preparation to stimulate the body’s immune response against diseases [1]. Classically, weak and inactive pathogenic culture or some part of microbes is inoculated in the body to develop an immune response against a particular illness [2]. On one side, the immune system gets activated by the pathogenic particle of the vaccine, and resultantly the antibodies are developed against the invaded pathogens; this is known as an active immune response. On the other hand, certain vaccines contain readymade antibodies, termed a passive immune response [3].

Despite the devastating effects of SARS-COV-2 worldwide, there is a dire need for approaches to limit the spread of the virus, necessitating the need for prevention through vaccines. The World Health Organization (WHO) has already warned that more outbreaks might be seen and the only way to deal with this pandemic is immunization [4], and people around the globe are afraid of COVID-19 and its upcoming variants [5].

Globally, the compromised state of immunity is a great peril during the ongoing pandemic crisis. Especially geriatric people are at greater risk due to compromised health. Many factors are involved in causing health hazards during COVID-19, such as hypertension, diabetes, obesity, and other comorbidities. These diseases are somehow related to the compromised immune response [6]. Consequently, already existing pulmonary infections may enhance the vulnerability to adverse events among vaccinated subjects of COVID-19 [7,8].

The response to vaccines is known to vary among individuals and can be influenced by a range of factors, such as vaccine administration techniques, storage conditions, and genotypic characteristics [9]. The prevalence of post-vaccine side effects is an essential consideration in the current pandemic. Various factors can contribute to the prevalence of those side effects, such as age, gender, and type of vaccine [10]. The precautionary measures in any vaccination therapy are crucial as the vaccine protects people against any harmful illness. However, adverse drug events might be shown over time [11–13]. Additionally, the fear of COVID-19 vaccines’ side effects is bothering the population’s minds to accept COVID-19 vaccines [14].

A vaccine has no doubt a benefit but based on the precedent studies on Middle East Respiratory Syndrome (MERS) and Severe Acute Respiratory Syndrome (SARS), researchers believe that vaccination therapy may amplify the disease severity in SARS-COV-2 [15,16]. On the other hand, it has been claimed that Chinese COVID-19 vaccination therapy is very effective and has no hazards [17]. Furthermore, Pfizer claims the effectiveness of their Vaccine (BioNtech®) is more than ninety percent; Moderna®, AstraZeneca®, and SinoVac® are more than seventy percent effective [17]. As per the report of WHO, the Sinopharm COVID-19 vaccine produced mild to moderate side effects among the recipients during the phase 3 clinical trial [18]. Therefore, this study aimed to determine the reported side effects among various COVID-19 completely immunized communities of Southern Pakistan.

## Material and methods

### Study design and data source

A questionnaire-based quantitative cross-sectional study was performed from August to October 2021 to analyze the side effects of various available COVID-19 vaccines in Southern Pakistan. An online survey link was only active until the desired sample size was achieved. The study is based on a self-designed questionnaire on Google forms distributed to the general population through social media. A simple random sampling technique was used for sampling, where the sample of this study was the population of southern Pakistan. Initially, the survey questionnaire was distributed through various WhatsApp and Facebook groups for the data collection. A mandatory informed consent statement was present at the start of the survey, and only those participants who agreed to participate were included. Therefore, this study included only participants above 18 years, residents of southern Pakistan, and fully vaccinated according to the COVID-19 vaccine schedule [19].

### Tool design and reliability

The tool was designed in the English language on google-forms, and its link was distributed to social contacts through social media. At the initial stage, the clarity was tested by distributing it to 50 participants who were excluded from the final evaluation. In order to assess the reliability of the questionnaire, Cronbach’s alpha test co-efficient was computed, resulting in a value of 0.78, which was deemed satisfactory according to established standards. The first portion at the opening of the link was about the consent, whether the individual was willing to participate or not. Only those participants who agreed to participate in the research had access to the further sections. The second portion included personal information such as age, gender, and information regarding COVID-19, if they have previously been exposed to it or not. The third section included comorbidity profiles of participants, and vaccine information, including the type of vaccine received. Lastly, the questions regarding side effects after the first and second immunization doses were included in the fourth section. Each respondent was required to report the side effects they experienced after getting the first and second doses of the vaccine separately. Moreover, each respondent was allowed only once to fill out the survey.

### Sample size

The sample size was calculated with a 95% confidence interval by an online Raosoft sample size calculator [19] at a 5% margin-of-error with a total vaccinated population of 25,493,964 as of September 20, 2021. The total population completely vaccinated was taken from the official website of the National Command and Operation Center (NCOC) [20]. The minimum sample size required was 385. A total of 602 responses were recorded; ninety-five respondents denied participation and hence were excluded. The response rate was 84.5%.

### Statistical analysis

Continuous variables were elaborated in mean ±SD. Categorical variables (age, gender, comorbidity, types of COVID vaccines) received were explained in frequencies and percentages (%). Chi-square **(***χ*^*2*^) test was performed to find the association among the categorical variables and check the significance level at *P < 0*.*05*. The age was divided into two groups (below 45 and above 45 years) ranging from 18 to 65 years. All the data analysis was performed on SPSS v.23.

### Bio-ethical approval

The Institutional Bioethics Committee (IBC) certificate was obtained from the office of Research, Innovation, and Commercialization (ORIC), University of Sindh, Jamshoro, Pakistan **(ORIC/SU/844).** An electronic informed consent at the beginning of survery form was included, containing answer by the participant as Yes or No, before commencement to the further sections.

## Results

A total of 507 responses were recorded. All the respondents were completely vaccinated.

### Demographic features and comorbidity status of respondents

Most respondents were male (n = 327; 64.5%). The mean ±SD age was 33.3 ± 8.5 years. Most respondents were under 40 (n = 464; 92.5%) (Table 1). Moreover, most respondents were free from comorbidities (n = 370, 73%). The frequently observed comorbid conditions were diabetes mellitus (n = 42; 8.3%), allergy (n = 38, 7.5%), and hypertension (n = 37; 7.3%). **Table 1** highlights all the demographic features of the respondents.

**Table 1:** Demographics of respondents.

Characteristics	N	%
**Gender**		
Male	327	64.5
Female	180	35.5
**Age**		
40 years	464	91.5
>40 years	43	8.5
**Comorbidity**		
Yes	137	27
No	370	73
**COVID-19 history**		
Yes	344	67.9
No	163	32.1
**Type of Vaccine**		
CoronaVac	126	24.9
BBIBP-CorV	185	36.5
BNT162b2	72	14.2
AZD1222	70	13.8
mRNA-1273	54	10.7
**Comorbidities**		
No Comorbidity	370	73
Hypertension	37	7.3
Allergy	38	7.5
Cardiovascular disease	4	0.8
Asthma	16	3.2
Diabetes Mellitus	42	8.3

### COVID-19 and vaccination history of respondents

The majority of respondents had a positive history of COVID-19 (n = 344, 67.9%). Most respondents were immunized with the BBIBP-CorV vaccine (n = 185, 36.5%) and the CoronaVac vaccine (n = 126, 24.9%). **Table 1** illustrates the descriptive analysis of COVID-19 among respondents.

### Post-vaccination side-effects after the first dose of the COVID-19 vaccine

**Table 2** elucidates that the most prominent side effects reported after the first dose were body aches (CoronaVac 54.8%, p < 0.05; BNT162b2 61.1%, p < 0.05; AZD1222 48.6%, p < 0.05), fever (BBIBP-CorV 8.1%, p < 0.05; BNT162b2 26.4%, p = 0.05; mRNA-1273 53.7%, p < 0.05), weakness (CoronaVac 42.9%, p< 0.05; BNT2b2 26.4%, p < 0.05), lethargy (BBIBP-CorV 28.6%, p < 0.05; AZD1222 65.7%, p < 0.05) and pain at the site of injection (CoronaVac 83.3%, p < 0.05; BBIBP-CorV 62.2%, p < 0.05; BNT162b2 86.1%, p < 0.05).

**Table 2:** Side effects after the first dose of vaccination.

Side effects	Corona-Vac (126)	p-value	BBIBP-CorV (185)	p-value	BNT162b2 (72)	p-value	AZD-1222 (70)	p-value	mRNA-1273 (54)	p-value
Body ache	69 (54.8)	0.000	14 (7.6)	0.000	44 (61.1)	0.000	34 (48.6)	0.014	19 (35.2)	0.959
Fever	26 (20)	0.44	15 (8.1)	0.000	19 (26.4)	0.057	4 (5.7)	0.003	29 (53.7)	0.000
Flu	4 (3.2)	0.49	0	--	8 (11.1)	0.000	0	--	0	--
Headache	57 (45.2)	0.001	115 (62.2)	0.131	38 (52.8)	0.352	47 (67.1)	0.088	36 (66.7)	0.162
Chills	1 (0.8)	--	42 (22.7)	0.000	4 (5.6)	0.582	29 (41.4)	0.000	2 (3.7)	0.304
Weakness	54 (42.9)	0.000	5 (2.7)	0.000	19 (26.4)	0.009	3 (4.3)	0.004	0	--
Itching	0	--	5 (2.7)	0.951	4 (5.9)	0.118	3 (4.3)	0.402	2 (3.7)	0.655
Lethargy	9 (7.1)	0.000	53 (28.6)	0.015	4 (5.6)	0.000	46 (65.7)	0.000	3 (5.6)	0.001
Pain at the Injection site	105 (83.3)	0.002	115 (62.2)	0.000	62 (86.1)	0.005	47 (67.1)	0.291	38 (70.4)	0.726

### Post-vaccination side effects after the second dose of COVID-19

**Table 3** highlights that the prevalence of side effects after the second dose was higher than the first. The most prevalent side effects after the second dose includes; body ache (CoronaVac 66.7%, p < 0.05; BBIBP-CorV 27.6%, p < 0.05; mRNA-1273 50%, p < 0.05; AZD1222 14.3%, p < 0.05), headache (BBIBP-CorV 69.2%, p < 0.05; mRNA-1273 64.8%, p < 0.05), lethargy (BBIBP-CorV 49.7%, p < 0.05; mRNA-1273 55.6%, p < 0.05) and pain at the site of injection (CoronaVac 55.6%, p < 0.05; BBIBP-CorV 70.8%, p < 0.05; BNT162b2 54.2%, p < 0.05).

**Table 3:** Side effects after the second dose of vaccination.

Side effects	Corona-Vac (n = 126)	p-value	BBIBP-CorV (n = 185)	p-value	BNT162b2 (n = 72)	p-value	AZD-1222 (n = 70)	p-value	mRNA-1273 (n = 54)	p-value
Body ache	84 (66.7)	0.000	51 (27.6)	0.004	9 (12.5)	0.000	10 (14.3)	0.000	27 (50)	0.020
Fever	6 (4.8)	0.000	1	--	27 (37.5)	0.000	10 (14.3)	0.983	28 (51.9)	0.000
Flu	1 (0.8)	--	0	--	1	--	9 (12.9)	0.000	5 (9.3)	0.007
Headache	7 (5.6)	0.000	128 (69.2)	0.000	41 (59.9)	0.008	5 (7.1)	0.000	35 (64.8)	0.000
Weakness	1 (0.8)	--	0	--	0	--	0	--	0	--
Diarrhea	8 (6)	0.002	5 (2.7)	0.881	0	--	0	--	0	--
Chills	0	--	0	--	35 (48.6)	0.000	0	--	0	--
Lethargy	27 (21.4)	0.000	92 (49.7)	0.000	26 (36.1)	0.997	8 (11.4)	0.000	30 (55.6)	0.002
Nausea	0	--	0	--	8 (11.1)	0.000	0	--	3 (5.6)	0.071
Pain at the Injection site	70 (55.6)	0.811	131 (70.8)	0.000	39 (54.2)	0.931	6 (8.6)	0.000	31 (57.4)	0.665

### Side effects and presence of the comorbid condition

In order to know this, we divided the respondents into two groups- with comorbidity and no comorbidity. It was assessed that the prevalence of side effects was slightly more after the second dose. However, there was no marked significant association of side effects after the first and second doses due to the comorbid condition. **Table 4** describes the side effects after the first and second vaccination doses in the presence of a comorbid condition.

**Table 4:** Post-vaccination side effects and presence of the comorbid condition.

Side effects	After 1^st^ dose			After 2^nd^ dose		
	Co-morbidity (n = 131)	No comorbidity (n = 358)	p-value	Comorbidity (n = 137)	No comorbidity (n = 370)	p-value
Body ache	44 (32.1%)	136 (36.8%)	0.33	46 (33.6%)	33 (36.5%)	0.54
Fever	17 (12.4%)	76 (20.5%)	0.03	14 (10.2%)	58 (15.7%)	0.11
Flu	0	12 (3.2%)	0.03	9 (6.6%)	7 (1.9%)	0.007
Headache	82 (59.9%)	211 (57%)	0.56	50 (36.5%)	166 (44.9%)	0.09
Weakness	20 (14.6%)	61 (16.5%)		0	1 (0.3%)	0.54
Diarrhea	12 (8.8%)	30 (8.1%)	0.81	2 (1.5%)	11 (3%)	0.33
Chills	18 (13.1%)	18 (4.9%)	0.001	0	35 (9.5%)	0.000
Abdominal pain	--	--	--	13 (9.5%)	25 (6.8%)	0.29
Lethargy	48 (35%)	67 (18.1%)	0.000	46 (33.6%)	137 (37%)	0.43
Nausea	--	--		0	11 (3%)	0.04
Pain at the Injection site	93 (67.9%)	274 (74.1%)	0.16	61 (44.5%)	216 (58.4%)	0.005

## Discussion

The current study aimed to assess various side effects experienced by people after being vaccinated with different COVID-19 vaccines. This assessment was crucial to record the commonly observed side effects of COVID-19 vaccines. The analysis in our study showed that, after the first dose of vaccines CoronaVac had 335 side effects, BBIBP-CorV had 368 side effects, BNT162b2 had 204 side effects, AZD-1222 had 213 side effects, and mRNA-1273 had 129 side effects. Similarly, after the second dose, CoronaVac had 204 side effects, BBIBP-CorV had 408 side effects, BNT162b2 had 186 side effects, AZD-1222 had 48 side effects, and mRNA-1273 had 159 side effects.

Consistent with our findings, a survey from the United States reported mild adverse effects following COVID-19 vaccination [21]. However, eighty percent of the population developed post-vaccine side effects. Similarly, three million doses of AstraZeneca® were administered in the United Kingdom. It was observed by the safety and monitoring system of the United Kingdom that in every million administered vaccines, about four thousand cases show post-vaccination adverse drug reactions [21]. In our study, 96.8% of AZD1222 recipients developed post-vaccine side effects after the first dose of the vaccine, which was slightly higher than the mentioned study. Among the various AZD1222 recipients, body ache was prominent among 48.6%. In addition, fever was reported by 5.7%, headache by 67.1%, Chills by 41.4%, lethargy by 65.7%, and pain at the site of injection was reported among 67.1%. The possible reason behind this could be the triggered immune response due presence of adjuvants contained by AZD1222 [22,23].

In general, our study revealed that 35.5% of participants experienced body pain, 18.3% fever, 57.8% headache, and 72.4% felt pain at the site of injection. In line with our findings, the European Medicine Agency (EMA) reported that half of the population felt localized pain at the site of vaccine administration, lethargy, and headache [21].

As per our study, 98.6% of BNT162b2 recipients had post-vaccine side effects after the first dose, and 91.7% had post-vaccine side effects after the second dose. The most common side effects after the second dose of BNT162b2, as per our study, were body aches at 12.5%, fever at 37.5%, headache at 59.9%, chills at 48.6%, lethargy at 36.1%, nausea among 11.1% and pain at the injection site among 54.2% respondents. In contrast, 50% of the BioNtech® recipients had side effects during clinical trials [24]. Furthermore, another study reported that only 8% of recipients of the second dose of the BioNtech® vaccine had no adverse effects [25], which was slightly higher in our results in which 8.3% of BNT162b2 recipients had no side effects after the second dose. The possibility of augmented side effects is mainly higher among individuals who never encountered COVID-19 [26].

It appears to be confounding where various vaccines against SARS-COV-2 are offered, each claiming its peak effectiveness. This claim creates a dilemma in the minds of ordinary people regarding the selection of vaccines, consequently leading to vaccine hesitancy. Studies have revealed mild side effects of BioNtech®, Moderna®, and AstraZeneca® among the vaccinated healthcare providers in Germany. It was reported that among the mRNA-based vaccines, more than 50 percent had lethargy and headache, more than 30 percent had body pain, and more than 20 percent had body chills, arthralgia, and weakness [27]. Our study reported that among the recipients of the mRNA-1273 vaccine, 50% experienced body aches, 51.9% fever, 64.8% headache, 55.6% lethargy, and 57.4% pain at the injection site. Similarly, our results also revealed that among the AZD1222 recipients, 48.6% had body aches, 67% had headaches, and 41.4% had chills after the first dose, which was higher than in another study [27].

Another study conducted by Czech healthcare providers revealed that a significant proportion of recipients of the BioNtech® COVID-19 vaccine experienced various side effects. These included pain at the local injection site (89.9%), lethargic condition (62.2%), headache (45.6%), and myalgia (37.1%). Dermatological issues were also reported by 5% of the participants [28]. In our study, 61.1% reported body pain, 52.8% had a headache, and 86.1% had pain at the site of injection after the second dose of BNT162b2. The enhanced immune system due to the vaccine can augment cytokine production, which increases the inflammation of various body and other tissues. Thus it can mediate adverse events after the second dose of the vaccine [23].

A study involving Turkish healthcare providers revealed that post-vaccination visceral and localized adverse effects such as headache (18.7%) and muscle aches (11.2%) were very commonly observed effects [29]. Similar studies investigated that post-vaccination pain, pyrexia, myalgia, and lethargy are very common mild adverse after the vaccination [30,31]. Moreover, in our study, the most common side effects were body aches, fever, headache, lethargy, and pain at the site of injection.

This study has elaborated on the COVID-19 vaccine-related side effects reported; however, there are certain limitations. The other vaccines, such as Ad5-nCoV vaccine recipients, were fewer in number and were excluded from the study. Therefore extensive studies in the future will be helpful in probing more about the side effects of vaccines.

## Conclusion

The side effects due to COVID-19 vaccination can vary between first and second doses and type of COVID-19 vaccine. The most commonly reported side effects were body ache, fever, lethargy, headache and pain at site of injection. Moreover, various factors are associated with possibility of post vaccine side-effects. Importantly, none of the vaccines caused immediate life-threatening side effects. Our findings suggest continuing monitoring vaccine safety and the importance of individualized risk-benefit assessment for COVID-19 immunization.

## Supporting information

S1 Data (XLSX)
